# Bryonin

**Published:** 1911-05

**Authors:** H. H. Redfield

**Affiliations:** Chicago, Associate Professor of Therapeutics, Bennett Medical College; and Professor of Active-Principle Therapeutics, Reliance Medical College (First chair of the kind established in America)


For Texas Medical Journal.

### Bryonin.

BY H. H. REDFIELD, M. D., CHICAGO,

Associate Professor of Therapeutics, Bennett Medical College; and Professor of Active-Principle Therapeutics, Reliance Medical College (First chair of the kind established in America).

Bryonin is a glucoside derived from bryonia alba (common name, white bryony), a plant indigenous to Europe. Dose of the standard granule, 1-67 grain.

*Toxic Action.*—The local application of bryonin is productive of blisters, and when taken internally in toxic doses it produces gastrointestinal inflammation accompanied by profuse vomiting and diarrhea. Mydriasis occurs, the temperature falls, there is colic, and death occurs, preceded by collapse.

*Therapeutics.*—In contradistinction to anemonin, which finds its best field of usefulness among blonds, bryonin seems to be especially indicated in brunettes, those of a bilious disposition or of a rheumatic diathesis. These, as a rule, are irritable and easy to anger, but of a firm fiber and strong personality. There is great thirst and large quantities of water are consumed at long intervals. The patient complains of a tearing, stitching pain, which is made worse upon motion but is relieved by rest. Sometimes these patients become delirious, their fancies being a rehash of the affairs of business, even to the smallest details. They are very restless and can be kept in bed only with difficulty.

Headache, which may be due to some gastric disturbance, rheumatic or congestive in nature, is relieved by bryonin.

Women who suffer from headache after doing a washing or ironing in an overheated stuffy kitchen, or as a result of constipation, find relief from bryonin. The pain usually is located in the forehead, is dull and throbbing in nature, and every exertion tends to increase it. Stooping, sneezing or coughing makes the pain more severe.

Cases of rheumatism, when the joints are red and swollen and the slightest motion produces the characteristic lancinating pains, speedily show improvement under bryonin.

In fevers, when the patient smells sour, due to the perspiration, is weak and exhausted, and the erect position causes nausea or vertigo, bryonin is the remedy of choice.

In typhoid fever, when the tongue is dry and cracked, or fissured, and the lips are the same; when stools resemble burnt sienna in color and consistency; when there is great thirst and large quantities of water are taken at long intervals; when the urine is dark and scanty; when the patient is in a semicomatose condition, or drowsy and sleeping all day but wakeful or delirious during the night, bryonin should be studied, as it will give good results.

In malaria, when the chills commence at the extremities or at the lips, and there is great thirst at all stages of the attack, with a perspiration that is either sticky or oily or sour-smelling, bryonin in full dosage is indicated.

In all congestions of the serous membranes it is the remedy to rely on. It, therefore, is to be thought of in congestion of the brain, which is due to exposure to severe cold, constipation, or the suppression of a natural discharge. Headache is present, being of a bursting, splitting character, generally located across the temples.

Bryonin gives good results in pleurisy of the plastic form, but its exhibition after effusion has occurred will meet with failure. The pains of pleurisy, which are intensified by taking a deep breath, are relieved by this remedy. It makes an admirable combination with asclepidin in pleuritic conditions, and both should be given in full dosage.

In all of the catarrhal conditions of the respiratory tract, after aconitine has done its work, bryonin should be exhibited. Usually the patient complains of a feeling of soreness all over the chest, and especially behind the sternum; the cough is dry and racking, and may be accompanied by faint nausea. There is a constant tickling in the throat, and the sensation as of a substance there which can not be dislodged by coughing, the presence of which produces a constant desire to cough. The cough is worse at night, and the soreness is made worse by motion.

It is indicated in inflammation of the pleura characterized by stabbing, lancinating, stitching pains which are worse on motion, the patient being in a state of mental unrest. There is dyspnea, the cough is dry and hacking and painful in the extreme; tongue coated; lips dry; great thirst; liver torpid; pulse high-tensioned; urine scanty; bowels constipated.

This drug is a remedy that should always be at hand during the pneumonia season. Called to a case of pneumonia, the exhibition of bryonin every fifteen minutes in solution, together with aconitine, digitalin and veratrine, calomel and podophyllin to clean out the intestinal tract, and the intestinal antiseptics to keep it clean, and saturation with calcium sulphide, these measures will bring about a change that is speedy and satisfactory, the result being a revelation to those who have been pursuing the old line of treatment. In some of these cases it is better to drop the veratrine after twenty-four hours, in which time the pulse and temperature should be normal, giving strychnine arsenate to convalescence. Cases treated in this manner under the writer's personal observation have gone to recovery in from one to two weeks without the slightest sign of complications.

In gastralgia, when the patient complains of a heavy, dull feeling in the stomach, bryonin is useful. There may be nausea and vomiting or flatulence, and a history of these cases will usually bring forth the knowledge that the sufferer has been indulging in such things as welsh rarebit, or malt brews. Pressure over the epigastrium causes great pain in these cases.

In congestive states of the liver or in atrophy of the liver, when the jaundice is marked and the patient complains of a pain or distressed feeling in the region of the right shoulder, accompanied by more or less vertigo upon arising from a prone position, bryonin should be remembered in conjunction with chelidonin, berberine and collinsonin.

Hard, dry stools, dark-brown in color, with torpidity of the bowels and no inclination to bowel movement, are indications for bryonin.

People who suffer from diarrhea brought on by cold drinks or exposure to cold showers are benefited by bryonin.

Inflammation of the mammary glands due to too early weaning of the infant, when the breasts are swollen, red and hot and exceedingly tender, find a comforter in bryonin. In these cases it should be studied with phytolaccin and atropine or pilocarpine.

Bryonin is a remedy that deserves sedulous study, and it should be exhibited whenever its cardinal indications—pain worse from motion—are manifest.

